# Perceptions and Practices regarding Breastfeeding among Postnatal Women at a District Tertiary Referral Government Hospital in Southern India

**DOI:** 10.1155/2016/5430164

**Published:** 2016-10-20

**Authors:** Sowmini P. Kamath, Dikshy Garg, Mohd. Khursheed Khan, Animesh Jain, B. Shantharam Baliga

**Affiliations:** ^1^Department of Pediatrics, Kasturba Medical College, Mangalore, Manipal University, Manipal, India; ^2^Kasturba Medical College, Mangalore, Manipal University, Manipal, India; ^3^Department of Community Medicine, Kasturba Medical College, Mangalore, Manipal University, Manipal, India

## Abstract

*Background.* Breastfeeding is the optimal method for achieving a normal growth and development of the baby. This study aimed to study mothers' perceptions and practices regarding breastfeeding in Mangalore, India.* Methodology.* A cross-sectional study of 188 mothers was conducted using a structured proforma.* Results.* Importance of breast feeding was known to most mothers. While initiation of breast feeding within one hour of birth was done by majority of mothers, few had discarded colostrum and adopted prelacteal feeding. Mothers opined that breast feeding is healthy for their babies (96.3%) and easier than infant feeding (79.8%), does not affect marital relationship (51%), and decreases family expenditure (61.1%). However, there were poor perceptions regarding the advantages of breast milk with respect to nutritive value, immune effect, and disease protection. Few respondents reported discontinuation of breastfeeding in previous child if the baby had fever/cold (6%) or diarrhea (18%) and vomiting (26%). There was a statistically significant association between mother's educational level and perceived importance of breastfeeding and also between the mode of delivery and initiation of breast feeding (*p* < 0.05).* Conclusion.* Importance of breast feeding was known to most mothers. Few perceptions related to breast milk and feeding along with myths and disbeliefs should be rectified by health education.

## 1. Introduction

Breast milk is the most ideal and valuable food for the growing infant since it suffices most of the nutritional requirements if given adequately and in appropriate manner. Exclusive breastfeeding for the first six months of life followed by nutritionally adequate and safe complementary foods with continued breastfeeding up to two years of age or beyond is the recommended practice by World Health Organisation and American Academy of Pediatrics [1]. Initiation of breastfeeding as early as possible has become mandatory as per baby friendly initiative norms, because it serves multiple purposes to the growing baby, mother, and also the whole family. Breastfeeding significantly reduces mortality in neonatal sepsis, pneumonia, and diarrhoea which was emphasised in* Lancet's* “Child Survival Series” of 2003 [2]. The sole effective preventive intervention to decrease 13–15% of all child deaths is by implementing universalisation of breastfeeding. This when coupled with adequate complementary feeding would prevent 19% of all child deaths (Lancet 2003, 2005) [3].

However, anecdotal reports suggest that the incidence of breastfeeding is declining in almost all parts of the world probably because of increasing modernization, introduction of artificial feeds, and early initiation of complimentary feeds. This could be attributed to increasing educational levels, with mothers being more employed.

The infant mortality rate in developing countries was six to ten times higher in non-breast-fed infants in the first months of life [4]. By 2015, Millennium Development Goals (MDG-4) targets aim at halving the neonatal mortality and improving newborn health along with reduction of under-five mortality rates by two thirds, between 1990 and 2015. This can be achieved by adopting optimal breastfeeding practices as recommended by WHO, for example, breastfeeding to be initiated within half an hour of birth and exclusive breastfeeding to be continued for 6 months [5]. Hence continued support with counselling is mandatory to mothers during antenatal period and postnatal period.

Breastfeeding also is influenced by race, socioeconomic factors, and cultural and educational background of parents. Discarding colostrum and delayed initiation of breastfeeding due to various reasons are still practiced in few communities. Early and unnecessary introduction of top feeding in incorrect dilutions and in unhygienic pattern are also quite prevalent in many communities. The key to successful breastfeeding is continuous vigilance over infant feeding practice in the community for timely interventions, to ensure optimal growth and development in the infant. The current study was designed to explore the perceptions and practices related to breastfeeding among postnatal mothers. This information will be useful to policy makers for the formulation of future interventional programs.

## 2. Materials and Methods

After approval from the institutional research ethics committee, a cross-sectional study was conducted at neonatal division, district tertiary referral government hospital. With 95% confidence level, 85% power using 56% as the reference proportion for knowledge [6], and providing for 10% nonresponse rate, the sample size came out to be 188. Primiparous and multigravida mothers were randomly selected. Primiparous and multigravida mothers who breastfeed their babies (full term or preterm) who were delivered by either means, that is, normal vaginal, assisted, or caesarean sections, over one-month duration (June 2012) were included in the study. Mothers who had lost their babies and mothers whose babies required intensive care or were advised not to breastfeed due to medical reasons were not included in the study. A structured interview schedule was prepared to collect sociodemographic data, perceptions, and practices of mothers regarding breastfeeding. Data was collected by interviewing mothers (in their own language) using an interview schedule. The interview schedule was developed based on review of literature and taking inputs from few stakeholders. The questionnaire was validated for content by experts in pediatrics and community medicine and family health. The questionnaire was pilot tested among few subjects to check for ease of administration and understandability among the participants. Informed consent was obtained from all the mothers prior to the interview.

A five-point Likert scale was used to determine their perceptions on various aspects of breast milk and breastfeeding. Once responses were collected, this was collated into three-point Likert scale (disagree, neither agree nor disagree, and agree) for analysis. Knowledge and perceptions regarding qualities of breast milk were assessed. Mothers' perceptions on breastfeeding with respect to her comfort with breastfeeding, cost, its effect on care of other family members, and effect on marital relationship were also elicited and documented. Community's outlook towards breastfeeding was elicited using questions that included feeling of shyness among mothers while breastfeeding in public places, role of community nurses and medical staff in encouraging breastfeeding, duration of maternity leave, and facilitation of breastfeeding at work places. Infant feeding experience was also collected in multigravida mothers. Their practices towards continuation of breastfeeding for their previous baby during special circumstances, that is, while menstruating, during sickness in mother, and if baby had fever/cold, diarrhoea, and vomiting, were assessed.

Towards the end, after each interview the exact methodology of breastfeeding was observed in some interviewees who consented and interventions were done accordingly.

Data was entered in MS Excel and analysed using Statistical Package for Social Sciences (SPSS) software version 11.5. Descriptive statistics were computed, frequency and percentage of various responses were calculated, and results were described as percentages. Chi square test was used to find out the association of maternal education, parity, birth weight, maternal age, and mode of delivery with importance of breastfeeding and initiation of breastfeeding. *p* value less than 0.05 was considered statistically significant.

## 3. Results

A total of 188 postnatal mothers (88 primigravida and 100 multigravida mothers) formed subjects in this study. Their age ranged from 17 to 35 years; majority (90/188; 47.9%) were between 21 and 25 yrs. Almost half (47.87%) were educated till high school, and 27 (14.36%) had completed preuniversity and were graduates. Most of the mothers (51%) were homemakers, 5.3% were professionals, and the rest were manual labourers. Majority of the mothers (120/188, 63.8%) were from households with per capita income of less than Rs 1000 per month. The sociodemographic characteristics of the respondents are depicted in Table 1.

A total of 99 male and 89 female babies were delivered. Mode of delivery was vaginal and by caesarean section in 70.21% and 29.79%, respectively. The mean birth weight was 2.71 kgs (range 1.9–3.5 kgs) and mean gestational age was 38.6 wks (range 35–40.5 wks). Importance of breastfeeding was known to a total of 150 (79.7%) mothers. The source of information was mainly either from doctors/nurses during their antenatal visits (*n* = 60; 31.9%) or from their own mothers (*n* = 70; 37.23%) even before they had come to the hospital for delivery.

Most of the mothers (61%) started breastfeeding within 1 hour of delivery; 30 mothers started to breastfeed their babies after 4 hrs of delivery of which 25 had delivered by caesarean section. Ten percent of women reported to have discarded colostrum, due to the wrong myth related to it that it is harmful for infant's health. Prelacteal feeding with formula feeds, water, and so forth was adopted by 16% due to inadequacy of milk and because of priming by their relatives that milk production occurs only by day 3 after delivery. Exclusive breastfeeding in the current pregnancy for a period of 6-month duration was intended by nearly half (90; 47.9%) of participating mothers (Table 2). Further, another half (98; 52.1%) said they intended to feed exclusive breast milk to their child till 4 months of age.

Regarding their perceptions on breast milk qualities (Table 3), 37.3% (71 mothers) opined that it is nutritious, an equal number were noncommittal, and one-fourth of the mothers disagreed with its nutritious aspect. While majority (181, 96.3%) of the mothers felt that breast milk is healthy for their babies, only 32 (17%) mothers agreed that breast milk protects babies from diseases. Nearly half (93, 49.4%) of the mothers agreed that breast milk would encourage bonding between the mother and baby. Contraceptive advantage of breast milk was disagreed by 42.6% (80) mothers.

It is evident from Table 3 that more than three-fourths, 79.8% (150), of mothers agreed that breastfeeding is easier than giving infant feeding formulas and family expenditure was reduced by giving breast milk to infants as per 115 (61.1%) mothers. Nearly half, 48.1% (90), of mothers agreed that a lactating mother should not feel shy of breastfeeding in public places. Breastfeeding did not have any negative effect on marital relationship as per 96 (51%) mothers; however 24 (12.8%) mothers felt that it would affect their marital relationship. Encouragement towards breastfeeding by doctors/nurses and by community was opined by 96.8% (182) and 75% (141) mothers, respectively.

There were only ten mothers who were employed; seven of them agreed that a period of three months of maternity leave would be sufficient for breastfeeding. Seven of them were satisfied with the designated areas for breastfeeding at their workplace.

With respect to previous pregnancy, when multigravida mothers were questioned regarding exclusive breastfeeding, 34%, 57%, and 9% had exclusively breastfed their babies for 1–4 months, 5–8 months, and 9–12 months, respectively. Half of them had continued breastfeeding for a total of 17 to 24 months in addition to administering complimentary feeds. Fifteen of these multigravida mothers had resorted to artificial feeding for their previous child, since they felt that it would make their children healthier.  Table 4 describes their attitude regarding continuation of breastfeeding during special circumstances; whatever circumstance they were questioned about, it was found that most of these multigravida mothers continued breastfeeding indicating few of them still do have misconceptions about continuing breastfeeding in the mentioned circumstances and they need to be educated.

At the end of the interview with the mother, the exact methodology of breastfeeding was observed in 97 mothers who had consented for the same. The factors assessed were position of the baby, position of the mother, latching at the breast, pattern of breastfeeding, and milk let-down reflex. Responses were documented as appropriate and inappropriate (Table 5).

## 4. Discussion

Poor feeding practices during the first 6 months of life attribute to 53% of acute pneumonia and 55% of diarrheal deaths as per WHO [7]. As per WHO estimates, in 2009, suboptimal breastfeeding, especially nonexclusive breastfeeding in the first 6 months of life, resulted in 1.4 million deaths and 10% of the disease burden in children younger than 5 years of age [8]. In this study, the importance of breastfeeding was known to 79.7% of mothers prior to delivery and this knowledge was imparted to them by doctors/nurses during antenatal visits (40%) and by their own mothers (46.7%). This is in contrast to various studies reported earlier [6, 9]. Increased awareness in our mothers could be partly due to increased education levels in this district (literacy rate in Dakshina Kannada district is 88.62% as per Indian Census 2011) [10]. As per Edmond et al. if all women initiated breastfeeding within 1 hour of birth, 22% of the infants would be saved from death [11]. In India, this would translate to saving 2,50,000 newborns from mortality. There was 2.4-fold increase in risk, if there was delayed initiation of breastfeeding. In South Asia, rates of first-hour breastfeeding vary from 23% (India) to 75% (Sri Lanka). Breastfeeding initiation within an hour of birth has shown to reduce infection specific neonatal mortality, independent of the effect of exclusive breastfeeding during the first 6 months of life [7].

In the present study, initiation of breastfeeding within one hour of delivery was done by 61% mothers which is higher than other studies ranging from 23.7 to 54.5% [6, 12–14]. Initiation of breastfeeding after 4 hours in babies delivered by caesarean section was carried out by nearly 16% mothers since mothers required postoperative care. Colostrum feeding was done by 90% of mothers and this rate is higher than studies by Bhanderi and Choudhary [14] and Singh et al. [15]. Previous studies on prelacteal feeding have depicted varying degrees at 38.1%, 51.7%, and 53%, respectively [12, 13, 15]. However in our study, it was adopted by 16% mothers. In our study there seems to be increased awareness of importance of colostrum and also there is decreased usage of prelacteal feeds.

A total of 98 (52.1%) and 90 (47.9%) mothers had intended to continue breastfeeding for a period of 4 and 6 months, respectively, as exclusive breastfeeding in the present study (Table 2). NFHS 3 survey on exclusive breastfeeding in India documented that 46% of babies are exclusively breastfed for the first six months of life [16]. Since 1990, the global rates on exclusive breastfeeding have remained stagnant with only 37% of children younger than 6 months being exclusively breastfed despite repeated emphasis on this aspect [17]. Also, there is little sign of improvement globally with early initiation of breastfeeding at 43% and continued breastfeeding from 20 to 23 months at just 55% [18]. While there is some literature depicting benefits of exclusive breastfeeding even if done only till 3 months of age, exclusive breastfeeding only till 3 months is not an ideal solution in India and is not to be encouraged or routinely recommended.

In our study, of the 100 multiparous mothers, 51% and 35% of them had breastfed their previous child for 9–16 months and 17–24 months, respectively. Fifteen mothers had resorted to artificial feeding, the reason being lack of breast milk in 5 mothers and that ten mothers felt that, by administering artificial feeds, their baby would become healthier. Poverty and ignorance force them to continue breastfeeding after 6 months as an exclusive measure which would lead the child towards malnutrition if complimentary feeding is not introduced after six months.

The idea that breast milk is healthy for their baby was the only quality (Table 3) which was appreciated by mothers. The other qualities of breast milk had dissatisfactory responses and this is discordant with study done in Ghana by Singh [19]. This indicates that expecting mothers have to be stressed on the beneficial qualities of breast milk during their antenatal visits. Other perceptions (Table 3) were similar to a study by Khassawneh et al. [20]. Perceptions towards breastfeeding with respect to community and family in special circumstances (Table 3) were similar to a study conducted in Jordanian women [20]. Hundred multigravida mothers reported that they continued breastfeeding in special circumstances (Table 4) to their previous baby, which is better than responses seen in study by Ekambaram et al. [6].

In our study, association of mothers educations level with importance of breastfeeding was found to be statistically significant (*p* = 0.034); however with respect to breastfeeding initiation it was not significant. This is in contrast to data reported by Vatsayan et al. [21] and Giovanni and coworkers [22]. Association between mode of deliveries (vaginal and caesarean sections) and breastfeeding initiation was found to be statistically significant (*p* < 0.001). There was no significant association of parity, birth weight, and maternal age and mode of delivery with initiation of breastfeeding. In general, the breastfeeding practices were good but the position of the mother while feeding as well as latching of the baby need improvement. Given the fact that the participants came from low socioeconomic strata and with low literacy levels, these results are encouraging and can definitely be improved upon.

## 5. Conclusion

Importance of breastfeeding, practices of early initiation and exclusive breastfeeding, and their perceptions towards breastfeeding were fairly satisfactory among mothers included in this study. In view of certain prevailing myths and wrong practices, it would be essential to counsel mothers during antenatal period, regarding breastfeeding, stress the advantages of breast milk, and also dispel the myths and disbeliefs in them.

## Figures and Tables

**Table 1:** Sociodemographic characteristics (total *n* = 188).

Characteristics	*n* (%)
Age	
<than 20 years	24 (12.8)
21–25 years	90 (47.9)
26–30 years	55 (29.3)
31–35 years	19 (10.1)

Religion	
Hindu	149 (79.8)
Muslim	24 (12.8)
Christian	15 (8)

Type of family	
Nuclear	39 (20.2)
Joint	149 (79.8)

Mothers education status	
Below high school	71 (37.77)
High school	90 (47.87)
Preuniversity and above	27 (14.36)

Family size	
1–3 persons	24 (12.8)
4–6 persons	69 (36.7)
7–9 persons	83 (44.1)
>9 persons	12 (6.4)

Total family income (in rupees^*∗*^ per month)	
<2500	7 (3.7)
2500–5000	96 (51.1)
5000–7000	58 (30.8)
>7000	27 (14.4)

Per capita income (in rupees^*∗*^ per month)	
<500	17 (9)
500–1000	103 (54.8)
1000–1500	57 (30.3)
>1500	11 (5.9)

^*∗*^1 US dollar = 68 Indian rupees.

**Table 2:** Breastfeeding practices among lactating mothers in the current pregnancy (*N* = 188).

Sl. number	Breastfeeding practices	*n* (%)
(1)	Initiation of breastfeeding	
	Less than 1 hour	115 (61.2)
	1–<4 hours	43 (22.9)
	More than 4 hrs	30 (15.96)

(2)	Discarded colostrum	
	(a) Yes	19 (10)
	(b) No	169 (89.9)

(3)	Prelacteal feeds	
	(a) Yes	30
	(b) No	158

(4)	Type of prelacteal feeds	
	Water	13 (43.3)
	Formula feeds	16 (53.3)
	Herbal tea	0
	Others (honey)	1 (3.4)

(5)	How long do you intend to exclusively breastfeed this child?	
	(a) 0–4 months	98 (52.1)
	(b) 0–6 months	90 (47.9)

**Table 3:** Perceptions on the various aspects of breastfeeding (*n* = 188).

Question	Disagree *n* (%)	Neither agree nor disagree *n* (%)	Agree *n* (%)
Qualities of breast milk			
Nutritious	48 (25.5%)	69 (36.7)	71 (37.7%)
Healthy	1 (0.5%)	6 (3.2)	181 (96.3%)
Protection from disease	54 (28.7%)	102 (54.3)	32 (17%)
Encourage bonding	11 (5.9)	84 (44.7)	93 (49.4%)
Contraceptive advantage	80 (42.6%)	106 (56.4)	2 (1.1%)

Lactating mother should not feel shy of breastfeeding in public places	62 (33.1%)	35 (18.7%)	90 (48.1%)

Breastfeeding is easier than infant feeding formula	73 (3.7%)	31 (16.5%)	150 (79.8%)

Breastfeeding has no negative effect on marital relationship	24 (12.8%)	68 (36.2%)	96 (51%)

Breastfeeding is good way to decrease family expenses	23 (12.2%)	50 (26.6%)	115 (61.1%)

Community encourages breastfeeding over feeding infant formula	7 (3.7%)	40 (21.3%)	141 (75%)

Doctors/nurses encourage breastfeeding	2 (1.1%)	4 (2.1%)	182 (97.8%)

**Table 4:** Breastfeeding practices in special circumstances by multigravida mothers during their previous pregnancy (*N* = 100).

Particulars	Per cent
*Did you continue breastfeeding in following conditions?*	

During sickness in mother	
Yes	90
No	10

While menstruating	
Yes	87
No	13

When baby has fever/cold	
Yes	94
No	06

When baby has diarrhoea	
Yes	82
No	18

When baby has vomiting	
Yes	74
No	26

**Table 5:** Methodology of breastfeeding (*n* = 97).

Methodology of breastfeeding	Appropriate	Inappropriate
Position of the baby (baby in supine position, lying on mother's lap)	78 (80.4%)	19 (19.6%)
Position of the mother (mother in sitting position with her one hand under the baby)	67 (69.1%)	30 (30.9%)
Latching at the breast (baby properly sucks mothers nipple)	58 (59.8%)	39 (40.2%)
Emptying one breast per feed	11 (11.3%)	86 (88.7%)
Milk let down	65 (67%)	32 (33%)
